# Environmental implications of future offshore renewable energy development in Aotearoa New Zealand

**DOI:** 10.1080/03036758.2024.2406829

**Published:** 2024-10-24

**Authors:** Rachel Hale, David Thompson, Tom Brough, Louise Kregting, Melanie Hayden (Ngāti Huia ki Poroutāwhao, Ngāti Raukawa ki te Tonga, Te Ātiawa ki Whakarongotai, Ngāti Toa, Ngāti Pākeha), Darren Parsons, Scott D. Nodder, Jennifer Beaumont, Owen Anderson, Craig Stevens

**Affiliations:** a National Institute of Water & Atmospheric Research, Nelson, New Zealand; b National Institute of Water & Atmospheric Research, Wellington, New Zealand; c National Institute of Water & Atmospheric Research, Dunedin, New Zealand; d The New Zealand Institute for Plant & Food Research Ltd, Nelson, New Zealand; e National Institute of Water & Atmospheric Research, Hamilton, New Zealand; f University of Auckland, Auckland, New Zealand

**Keywords:** Mitigation, offshore wind energy, renewable energy, impact assessment, seabirds, marine mammals, shelf seas, marine renewables

## Abstract

Global climate mitigation efforts seeking to reduce greenhouse gas emissions require more renewable energy generation and utilisation. In Aotearoa New Zealand there are initiatives underway to develop offshore wind, or in the future, arrays of tidal turbines or wave energy converters, as a new energy resource. Here we synthesise available knowledge from international developments in offshore windfarm installations and discuss in a local Aotearoa New Zealand context. Aspects described include habitat modification, consequences of physical water column changes, and effects on benthic organisms, fish and fisheries, seabirds and marine mammals. Importantly, there is a need to adhere to Te Tiriti o Waitangi which defines Māori sovereign rights and expectations in terms of guardianship of resources (kaitiakitanga). Based on recent regulatory applications in marine spatial planning, where developments have been subject to the precautionary principle for environmental impacts, comprehensive environmental information will be critical for obtaining approval to proceed. The present synthesis identifies environmental pressure-points, footprints, and knowledge gaps, such as New Zealand-specific seabird and marine mammal behaviour and discusses potential opportunities to leverage the positive impacts of marine renewable energy developments.

## Introduction

Despite the present relatively high proportion (∼85%) of electricity supply in Aotearoa New Zealand from renewable sources (e.g. hydroelectricity, onshore wind, solar and geothermal – MBIE 2023), the global climate emergency makes it clear there is a need for substantially more energy to be derived from such sources, rather than via the continued combustion of hydrocarbons (coal, oil and petroleum) (IPCC 2022). This is required to reduce energy supply vulnerability and enable conversion of as much of the non-electricity part of the national energy sector to renewable sources as is possible. This is encompassed in New Zealand government policy milestones such as Carbon Zero 2050. Consideration of the state of the Aotearoa New Zealand energy sector emphasises the large size of contributions from the transport sector, the modest degree of self-sufficiency, and the presently small contribution of wind generation to national energy budgets (MBIE 2023).

**Figure 1.**
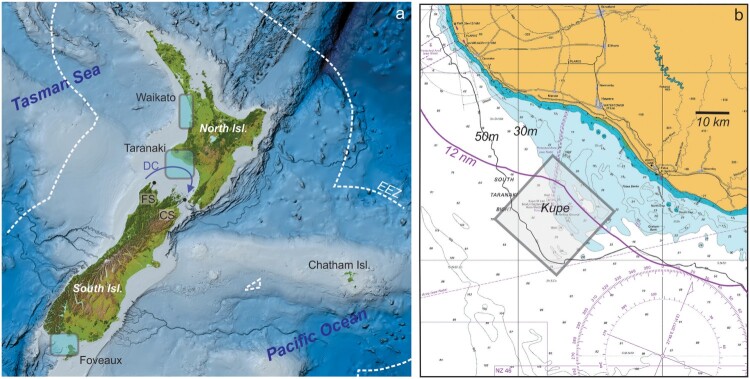
(a) A map of Aotearoa New Zealand and EEZ with key offshore wind development focal regions as well as the d'Urville Current (DC), Onetahua Farewell Spit (FS) and Te Moana a Raukawa Cook Strait (CS). Panel (b) shows the South Taranaki region (modified from excerpt from LINZ chart NZ48 Western Approaches to Cook Strait). Kupe is existing natural gas infrastructure, and the diamond is the general area of interest. Also shown is the 50 m depth contour and the 12 nautical mile limit, while the blue region is less than 30 m depth.

In 2022 the New Zealand Central Government initiated the development of a new marine/offshore renewables sector (MBIE 2022), motivated by the quality of the available resource (winds, waves, tides), a need for reduced social impact, and space availability in the marine environment. In doing so they identified target geographic regions (Figure 1) and the potential scale of required development. Beyond that, it was left to the sector to develop further activity and knowledge on resources, environmental conditions and potential impacts.

This Aotearoa initiative was motivated by the relative technological and economic certainty associated with the high level of international activity in offshore wind renewables. This has seen substantial growth of new marine renewable energy developments (MRED) in regions such as Europe, China and the USA. From the perspective of environmental impact, these jurisdictions have substantial histories of baseline data and assessment. Aotearoa New Zealand has historically had a well-evolved environmental impact assessment process for addressing the likely impacts of anthropogenic activities in the marine environment (New Zealand Government 2023a; see for example Clark et al. 2017). However, lack of environmental knowledge and available data have affected proposed activities in the New Zealand coastal ocean, such as seabed mining (Macpherson et al. 2021), and so development of improved understanding will be important in the present context of offshore energy (e.g. Macpherson et al. 2021). In addition, meeting the obligations of Te Tiriti o Waitangi (in part an agreement on how future resources would be shared and sustained, developed by Māori and European leaders as Aotearoa New Zealand’s founding partnership document in 1840) is another important aspect of any future developments in Aotearoa.

As reviewed in Dorrell et al. (2022), modern offshore wind energy capture activities typically take place in the inner continental shelf region, with scoping documents pointing to more remote offshore locations as being viable, likely to be beyond the current economic horizon. These deeper water applications will also require an advance in floating turbine technology (McMorland et al. 2022). Globally, this inner continental shelf zone has significant geophysical, ecosystem, societal, cultural, and economic values. Clearly, wind resources, operational infrastructure and environment interactions, will all have unique local aspects.

In the present paper, we synthesise available knowledge from international developments in the context of what is known locally from relevant past work in Aotearoa New Zealand. This is, by definition, not a systematic review of the entire topic because that requires actual installations and associated impact assessment in Aotearoa New Zealand waters. Instead, the synthesis is based on expert advice from a range of areas in the Aotearoa New Zealand applied marine science sector. Aspects described include habitat modification, impacts on benthic fauna, fish, seabirds, and marine mammals, and consequences of physical water column changes (e.g. turbulent mixing, stratification). We do not provide a review of mitigation measures or methodologies for addressing data gaps. While international work is, in many cases, directly relevant to the topic, there are of course many location-specific aspects of environmental impacts that need to be considered.

## Te Tiriti o Waitangi (The Treaty of Waitangi)

There is a critical contextual point whereby Aotearoa New Zealand differs from Europe, where the local energy transitions literature is most prevalent, because of the requirement to consider the rights, interests, and worldviews of the indigenous Māori people under the Treaty of Waitangi (MacArthur and Matthewman 2018; Kerr et al. 2015) – i.e. ‘The Treaty’ or ‘Te Tiriti’. Te ao Māori (a Māori worldview; see Table 1 for glossary of te reo (Māori) terms) is holistic. A general theme of te ao Māori is the interconnections between all things tangible and intangible, derived from whakapapa (common descent) (Cram et al. 2008). Traditionally, the environment was central to Māori society (Durie 1998). Māori health, wellbeing, and survival was dependent on the sustainability of the resources in the environment (Garven et al. 1997). This interdependence defines the relationship between Māori and the world, where Māori have a responsibility to manage their impact on other forms of life and ensure the survival of all into the future (Hauraki Trust Board 1999). This responsibility is known as the ethic of kaitiakitanga (guardianship, stewardship). Mana whenua and mana moana (people with authority over the land or sea) have a right to practice kaitiakitanga over their lands and waters.

**Table 1.** Glossary of te reo (Māori) terms.

Te reo	English
Aotearoa	The Māori-language name for New Zealand
Aukati	Cultural ban
Iwi	Tribe, nation, people
Kai, kai moana	Food, seafood
Hapū	Sub-section of iwi and the primary political unit
Kaitiakitanga	Guardianship
Kaupapa	Agenda, issue, strategy, plan
Mana	Authority
Mana moana	People with authority over the sea
Mana whenua	People with authority over the land
Moana	Ocean, coast
Rohe	Region, area
Tangata whenua	The Māori people of a particular region, or as a whole the original inhabitants of New Zealand
Taonga	Treasure (including social)
Te ao Māori	Māori worldview
Te reo Māori	The Māori language
Te Tiriti	The Treaty of Waitangi: Aotearoa New Zealand’s founding document
Wahi tapu, wahi taonga	Restricted sites, sites of treasure
Whanau	Extended family group
Whakapapa	Common descent

MRED installation and maintenance changes the functioning, aesthetics, economics, and social interactions of the coastal environment leading to potential conflicts between new MRED-related activities and traditional maritime human activities. For example, MRED construction can reduce access to traditional fishing grounds forcing a displacement of fishing activities with potential to cause economic loss and impacts on coastal communities (Stelzenmüller et al. 2021). These impacts have often been neglected in previous scientifically focussed MRED planning activities (Gee and Burkhard 2010; Busch et al. 2011) but are necessary in Aotearoa New Zealand under Te Tiriti and are essential to consider as part of maritime spatial planning.

Environmental sustainability and sustainable social, cultural and economic development have become important considerations for many, if not all, iwi and hapū (Cram et al. 2008; hapū: sub-section of iwi and the primary political unit; iwi: tribe, nation, people). It is therefore important when developing offshore renewable energy solutions, to consider environmental impacts in conjunction with cultural impacts. Generally, iwi and hapū are looking to support developments away from offshore oil and gas exploration and offshore renewables could present good alternative energy sources. Te Uri o Hau, hapū of Ngāti Whātua based in the Kaipara Harbour in northern Aotearoa New Zealand state within their environmental management plan: ‘from an environmental perspective, wind energy is one of the best renewable generation options immediately available in Aotearoa New Zealand. Fossil fuel alternatives emit greenhouse gases, but wind farms are a sustainable and environmentally responsible alternative for electricity generation’ (Te Uri o Hau Settlement Trust 2011).

However, the holistic view that Māori have of the marine environment means MRED cannot be considered in isolation of the wider ecosystem. For example, in the Kaipara Harbour, Te Uri o Hau objected to the development of tidal energy turbines due to the risk that the implementation of these turbines had to the already fragile harbour ecosystem (Bargh 2014). While the hapū did not object to renewable energy in principle, the ‘tribal values of environmental guardianship of the harbour and its ecosystem, which they see as a treasure that should be protected, means that despite the energy production being renewable, it is not viewed as appropriate’. Although the tidal energy development was consented in Kaipara Harbour by the regional council, the hapū placed an ‘aukati’ (cultural ban) over the developer company, and the initiative did not go ahead (Kerr et al. 2015). After the fact it became clear that the technology was not mature enough anyway (Stevens 2024).

## Impacts

Impact of any activity in the natural environment will occur over the infrastructure development lifecycle, including the: (i) exploration, (ii) installation, (iii) operation and (iv) de-commissioning phases. The nature of the impacts will change over these phases, both spatially and temporally. Given that, at the time of writing, there hve been no MRED installations, beyond sea trials, in Aotearoa we need to infer the environmental interactions from international studies (Whiting et al. 2019), as well as from local activities such as aquaculture, natural oil and gas exploitation, and seabed mining. All of these pursuits have had either a history of activity or assessment/evaluation in the New Zealand marine environment.

### Location

The New Zealand Government initiative identifies three regions for wind energy development (Figure 1): (i) South Taranaki Bight, (ii) western Waikato coast and (iii) Te Ara a Kiwa Foveaux Strait – with emphasis on the Taranaki region. All regions are suitable in terms of wind resource as Aotearoa is relatively windy in terms of global comparative metrics, having about double the global average wind capacity factor (Zhang et al. 2023). The South Taranaki Bight region was one of the first marine regions in New Zealand to be assessed for environmental risk to anthropogenic activities in the 1970s and 1980s, with the development of a local offshore oil and natural gas industry (The Maui Development Environmental Study – e.g. Bowman et al. 1983). The Kupe Natural Gas Field is co-located with the identified offshore wind development area (Figure 1). This is not entirely a coincidence as there is a need to identify a future pathway for a ‘just transition’ if New Zealand is to move equitably to a low-emission society. Seeking to enable the Taranaki region to shift from a fossil-based economy to renewable energy fits within that just transition framework. Here we focus somewhat on the Taranaki region but maintain wider comments where relevant to the other locations, or more generally around the 12 nautical mile Territorial Sea and the 200 nautical mile Exclusive Economic Zone (EEZ) (Figure 1). This domain could evolve with future development of floating turbine technology but for now is consistent with present focus (McMorland et al. 2022).

### Benthic effects

MRED can directly affect the benthic environment and provision of ecosystem services in coastal areas (Van de Pol et al. 2023). In some cases, the development of MRED has been a net positive for local biodiversity at multiple trophic levels (Lindeboom et al. 2011). However, knowledge of the effects of offshore renewable infrastructure development on the benthos is limited and benthic sensitivity to these activities may be higher than previously anticipated depending on local conditions (Dannheim et al. 2019). Most information on effects of ocean renewable infrastructure, particularly windfarms, on the local benthos currently exists for the construction and operational phases of the projects, although de-commissioning effects are likely to be comparable to those during the construction phase (Bergström et al. 2014; Dannheim et al. 2019; Lemasson et al. 2022). Potential effects of MRED during the infrastructure lifecycle include physical seabed disturbance (Miller et al. 2013), such as the removal of habitat through piling and cable trenching, sediment resuspension, changes in bathymetry and sediment type (Van den Eynde et al. 2013), noise, vibration, and electromagnetic field generation effects (Hutchison et al. 2020), metal pollution (Wang et al. 2023), reductions in light penetration to the seabed, and benthic habitat alteration (van Deurs et al. 2012; Herbert-Read et al. 2022; Wang et al. 2023), including the provision of new hard substrate from turbine piles and foundations (e.g. Vaissière et al. 2014). These potential effects can be expected during the lifespan of MRED, but effects and intensity may differ during particular phases (e.g. sedimentation and benthic disturbance during construction and decommissioning may be greater) and interactions of some of these impacts are not well known (Galparsoro et al. 2022).

These impacts could result in a reduced diversity of species and habitats which could have implications for sensitive environments (schedule 6 of the EEZ and Continental Shelf Act; New Zealand Government 2023b), protected species (schedule 7 of the Wildlife Act 1953; New Zealand Government 2022) and ‘At Risk’ or ‘Threatened’ taxa in the region (New Zealand Threat Classification System NZTCS – for details on categories see Townsend et al. 2008, updated by Michel 2021; invertebrate categories in Funnell et al. 2023)

A range of secondary effects may be important such as the seasonal timing of the activity, the foundation types of offshore MRED (Horwath et al. 2021), the species that interact with the installed infrastructure, and the intensity, duration, and severity of the benthic disturbance (Baulaz et al. 2023). However, depending on the substrate type, infaunal community composition could be robust to disturbance associated with offshore windfarm development activities (Degraer et al. 2020). Infaunal communities in sandy sediments may recover from disturbances more quickly those in muddier ones (Kaiser. 1998; Ferns et al. 2000; Dernie et al. 2003). After dredging of offshore sandbanks in the Belgian part of the North Sea for MRED construction, recovery of the infaunal community was observed after just one year (Coates et al. 2015). The soft substrate of the focal regions in Taranaki, Waikato and Te Ara a Kiwa Foveaux Strait comprises low mud (< 20%) and high sand (> 60%) (Bostock et al. 2019) and therefore infaunal communities may recover relatively rapidly from disturbances. The South Taranaki Bight is also a highly dynamic area with naturally high disturbance rates. The presence of an infaunal community already adapted to high levels of disturbance may mean benthic species composition and abundance is robust to MRED related disturbances and sedimentation (see Cummings et al. 2020).

Noise, vibration and electromagnetic fields from infrastructure installation and operation can affect marine epibenthos and infauna in a number of ways. Organism mortality (Kowalewski et al. 1993), physiology (Solan et al. 2016), behaviours such as species movements (Hutchison et al. 2018 and 2020; Scott et al. 2018; Van de Pol et al. 2023), responses to detrimental stimuli (Roberts et al. 2016), larval settlement and development (Pine et al. 2014), predator avoidance and foraging (Hughes et al. 2014) and bioturbation (Mosher 1972; Solan et al. 2016) may all be affected. However, the specific effects on many species (and life stages) are undocumented (Roberts and Elliott 2017), meaning many potential behavioural and mitigation strategies are unknown (Dannheim et al. 2019). This is particularly true in New Zealand waters where infaunal behaviours are, in general, not well known (Lam-Gordillo et al. 2023).

New Zealand places a high value on the exclusion of non-indigenous species (NIS – i.e. species that have been introduced to New Zealand with human assistance). They can degrade environmental, socio-cultural, and economic values (Hatami et al. 2022). In the marine space, these species are predominantly introduced via ballast water discharge and biofouling of hulls (Hatami et al. 2022). Novel marine habitat provision via MRED structures provide additional substrates for settlement and establishment of epifaunal communities (De Mesel et al. 2015; Coolen et al. 2016; Nall et al. 2017) while additional shipping activity in relation to construction and operation of renewable infrastructure can provide the conduit for introducing NIS (Dannheim et al. 2019). MRED infrastructure may also provide a stepping-stone of hard substrate for intertidal species between the non-indigenous species source and New Zealand shores as new habitat is provided in previously uninhabitable regions (Adams et al. 2014; De Mesel et al. 2015; Kerckhof et al. 2016; Bray et al. 2017).

Foundations, scour protection, turbine shafts, and other support structures provide substrate on the benthos, and throughout the water column, of varying composition and complexity, and can increase local biodiversity of invertebrate species (e.g. Petersen and Malm 2006; Andersson and Öhman 2010; Langhamer 2012; Vaissière et al. 2014). Indeed, this provision may double local species richness and increase benthic species abundances by two orders of magnitude (Li et al. 2023). These structures are generally installed into soft-bottom environments (Dannheim et al. 2019) where comparable natural hard surfaces are mostly absent. This provides a completely new habitat type for colonisation (Glasby and Connell 2001; Degraer et al. 2020). The addition of hard substrates in these environments may also increase organic enrichment in soft sediments close to MRED infrastructure due to deposition of shell debris and living and dead organic material originating from colonising fauna on the infrastructure (De Borger et al. 2021) leading to increased benthic species biomass and diversity surrounding these structures (Coates et al. 2014; Lefaible et al. 2023). In regions where species biodiversity is relatively low (e.g. Taranaki and Waikato: Stephenson et al. 2023), MRED could provide a significant boost to local biodiversity and primary production (Slavik et al. 2019). However, many of the taxa that establish on the new hard structures provided by MRED or the enriched benthos will be different from that of the natural ecosystem (Jouffray et al. 2020).

However, as commercial, recreational, and customary fishing could be restricted or even prohibited within MRED areas, they could operate as a de facto marine reserve leading to elevated biodiversity and productivity (Lefaible et al. 2023). Removal of direct fishing pressure and reduced trawling intensity, which can result in chronic disturbance of the benthos, may lead to benthic community changes and increased benthic biomass (Hale et al. 2017; Roach et al. 2018). Shifts in fish diets after MRED installation indicate a change in prey composition with increased occurrence of hard substrate prey species in the vicinity of offshore windfarm foundations (Raoux et al. 2018; Wilber et al. 2022; Buyse et al. 2023). Local soft sediment species could therefore benefit from the presence of MREDs through predation reduction via dispersion between greater benthic biomass availability (Buyse et al. 2023).

### Water column effects

**Figure 2.**
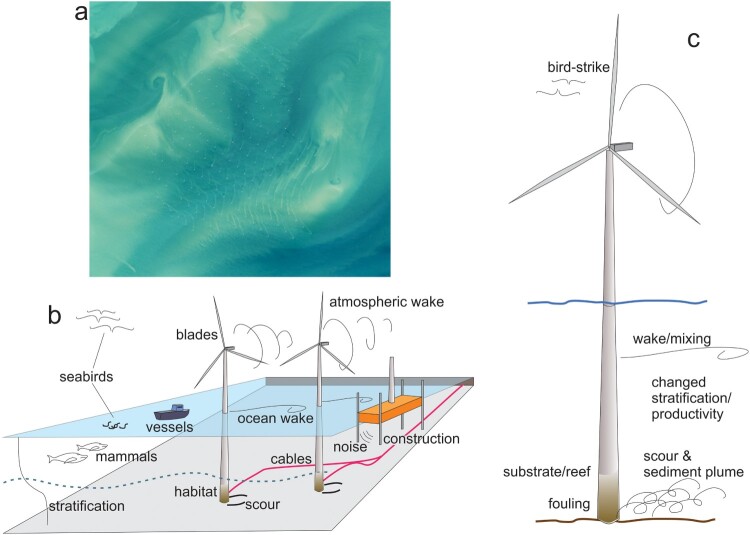
Multiscale synthesis (a) Sediment plumes from a wind turbine array in the English Channel captured by Landsat – the image is ∼15 km across (landsat.visibleearth.nasa.gov/view.php?id = 89063) (b) Sketch showing selected processes and issues. (c) Individual pylon-scale processes.

The presence of structures extending through the water column from the seafloor to the ocean surface results in hydrodynamic drag and mixing in the local environment (Figure 2). The mixing of the water column and associated effects on physical processes, such as vertical stratification, is an aspect of MRED marine environmental impacts that may have far-reaching effects (Clark et al. 2014; Paskyabi 2015; Øijorden 2016; Floeter et al. 2017). As Dorrell et al. (2022) state: ‘For the first time planned developments of both fixed and floating offshore wind infrastructure will add large scale anthropogenic mixing to seasonally stratified shelf seas’. While the primacy is debatable as large aquaculture installations generate similar effects (Plew et al. 2005), the ultimate impact is still true. The distributed array of crop in offshore aquaculture facilities creates drag which drives changes in local scale currents (Stevens et al. 2008). This will also affect erosion patterns around structures where scouring may lead to the preferential winnowing of fine sediments, resulting in a coarsening of the sediment substrate and causing changes in biogeochemical processes which can impact local long-term carbon storage potential in marine sediments (Pratt et al. 2014, Christiansen et al. 2022) and the ecological functioning of seafloor communities (De Borger et al. 2021). Changes such as the decrease or even disappearance of stratification due to locally induced turbulence may result in an upward transport of nutrients, affecting both local primary production (van der Molen et al. 2014; Floeter et al. 2017), and carbon flow to the benthos (Dannheim et al. 2019).

In New Zealand, a number of studies of shelf seas biophysical processes resulted from the initial development of the 1980s gas fields in offshore Taranaki (e.g. Bowman et al. 1983). More recently, Chiswell et al. (2017) examined transport of nutrients in the western Greater Cook Strait including the Taranaki focal region as identified in the MBIE (2022) report on MRED. The dominant physical ocean feature in the region is the D’Urville Current which flows to the northeast from around Onetahua Farewell Spit towards the Taranaki peninsula before turning southeast to flow through Te Moana a Raukawa Cook Strait into Te Moana-nui-a-Kiwa Pacific Ocean (Stevens et al. 2021). Associated with this oceanographic feature is the episodic Kahurangi Upwelling, which is important in nutrient supply to the surface ocean with consequent effects on regional planktonic and fish ecosystems (e.g. Bradford and Chapman 1988; Bradford et al. 1993). The South Taranaki focal region (MBIE 2022) is located essentially where the D’Urville Current encounters Te Ika-a-Māui North Island. The presence of an array of structures nearby is unlikely to affect something as large as regional ocean currents; however, it might have a local influence on current trajectory (Plew and Stevens 2013; Raghukumar et al. 2023) and other physical processes, such as turbulence, mixing and stratification (e.g. Dorrell et al. 2022).

The downstream wakes from offshore installations will have an effect on nutrient supply, and hence biological productivity, with flow-on impacts to various trophic levels and ultimately to the benthos. These impacts will be largely connected to changes in suspended sediment and nutrient levels due to sediment resuspension and modifications of water column stratification (Floeter et al. 2017; Daewel et al. 2022; Dorrell et al. 2022). It is challenging to separate turbine effects from the natural physical or environmental variability and in some instances, natural variability will be greater than the impact (Schuchert et al. 2018). For example, Chiswell and Stevens (2010) look at larval trajectories in the wake of a large island to the south of the Taranaki region. This illustrates the scale of variability and flow-object interaction.

Generally however, the consulted studies all identified effects in the order of 10% in terms of biological productivity. An important point is the overall biophysical effect of turbines changes moving from very shallow (∼ 10 m) nearshore locations to deeper water on the continental shelf (Dorrell et al. 2022). The coastal shallows are typically well-mixed; however, in water deeper than 20 m stratification starts to control biological productivity. The physical disturbance of turbine structures will reduce this stratification over a region larger than the array scale (Dorrell et al. 2022), and thus altering water column productivity (e.g. van der Molen et al. 2014; Øijorden 2016; Slavik et al. 2019).

In terms of understanding how marine activities could impact such physical processes it is useful to consider the Trans-Tasman Resources Ltd application to mine the seabed for iron sand in the same South Taranaki region. While still under consideration, this application was unable to convince a court (or the New Zealand Environmental Protection Agency) that there was sufficient certainty around environmental impacts over the lifetime of the seabed mining activity to allow approval (MacPherson et al. 2021). There was an emphasis on the water column effects of a mining-induced turbidity plume and how this might affect both water column and benthic life. A key aspect of the water column impact was the footprint of disturbance which was not simply a function of the immediate area of activity, but was instead affected by ocean flows and dispersion over a much larger spatial domain. Saying that, the term ‘footprint’ is complex in this application as it can refer to: (i) ephemeral changes in the water column (Stevens et al. 2008), (ii) a relatively static benthic region (Miller et al. 2013) or (iii) an all-encompassing social and/or carbon perspective (Kaldellis and Apostolou 2017).

### Fish impacts

In terms of fish and fishing, wind farm structures may act as artificial reefs and so increasing the abundance of hard structures is likely to attract a range of fish species (Grossman et al. 1997; Langhamer 2012; Raoux et al. 2017). In terms of impact and benefit, the fish species that are boosted in abundance at these sites may differ to those previously found there, with changes likely in demersal fish species density, community composition, and richness (Abramic et al. 2022; Li et al. 2023; Van de Pol et al. 2023). Although fisheries may see a spillover effect, with emigration of juvenile and adult fish from the protected MRED area (Di Lorenzo et al. 2020; Halouani et al. 2020) these may be different to those species that previous fisheries were based on. However, aggregating fish can lead to overexploitation if fisheries are not properly regulated. This is likely to be controlled in a wind farm context as some fishery types (e.g. trawling) will not be able to operate inside wind farms even if there are more fish than before the marine installation. While the abundance of fish may increase around the structures (which may or may not lead to overexploitation of aggregated fish populations), it is often not clear if these higher abundances are either the result of aggregating existing fish around structures (with an associated decrease in fish abundance outside of the protected MRED area), or whether the structure actually boosts the productivity of the system (Williamson et al. 2019). This remains an open question in the literature on using artificial reefs for fisheries (Twigg et al. 2020). This is a shared-use issue rather than an environmental effect and so beyond the present scope. However, there are opportunities for improved outcomes with a collaborative approach to marine space usage (Stelzenmüller et al. 2021).

In an Aotearoa context there are impact and use parallels with green-lipped mussel aquaculture farms (Christensen et al. 2003) and also there has been substantial work in an Aotearoa context on mussel farms and fish (Underwood and Jeffs 2023). These artificial environments have much higher abundances of snapper resulting in significantly enhanced recreational fishing activity. For example, the Coromandel mussel farms are now the most popular domestic recreational fishing location and account for about 250 tonnes of snapper catch a year. A mussel farm has the additional attractant of the mussels themselves, but wind farm structures would develop their own fouling community and potentially function in a comparable way (e.g. Slavik et al. 2019).

Electromagnetic fields (EMFs) from undersea power cables can have behavioural effects on elasmobranchs, which can be attracted to EMFs as far as 250 m from cable routes (Hermans et al. 2024) and result in increased foraging distances (Hutchison et al. 2018). Anadromous fish species such as galaxiids and anguillid eels use earth’s magnetic field for navigation; the effect of EMFs on such species is poorly known but there is considerable potential for disruption to migratory activities of such species (Westerberg and Lagenfelt 2008; Hutchison et al. 2020). Significant injury or mortality to some fish species is also likely to occur through noise and vibration associated with each stage of the MRED lifecycle, especially the construction phase (Popper et al. 2023). The ability of fish to communicate, forage, and detect predators may also be affected (Mooney et al. 2020).

### Marine mammal impacts

Aotearoa New Zealand is an international hotspot for marine mammals, with more than half of the world’s species being found in our waters (Baker et al. 2019). New Zealand waters are home to several threatened taxa including nationally critical and endangered endemic species (e.g. Hector’s and Maui dolphin, New Zealand sea lion; NZTCS; Baker et al. 2019). In addition, they are a migratory corridor for the Oceania population of humpback whales (Constantine et al. 2021), provide nursery habitat for southern right whales (Torres et al. 2017) and contain important blue whale foraging grounds (Barlow et al. 2018). Each of the aforementioned whale populations were among the most heavily exploited in the era of industrial whaling and are only recently showing signs of recovery (Prickett 2002; Branch et al. 2004; Gibbs et al. 2018). A wealth of poorly known, ‘cryptic’ species occurs in New Zealand including deep-water and oceanic specialists (Baker et al. 2019). In New Zealand, all marine mammals are protected by the New Zealand Marine Mammal Protection Act (1978) and the Wildlife Act (1953) and are of immense importance for tangata whenua (the Māori people of Aotearoa New Zealand). Thus, along with demonstrated impacts on marine mammals from MRED internationally, there should be considerable emphasis on managing risks to these species in New Zealand waters.

The occurrence and severity of impacts from MRED on marine mammals has been shown to be highly species – and location-specific (e.g. Blackwell et al. 2004; Thompson et al. 2010; Brandt et al. 2011). Internationally, most studies have focussed on bottlenose dolphin, harbour seal, and harbour porpoise (Carstensen et al. 2006; Brandt et al. 2011; Russell et al. 2014; Vallejo et al. 2017; Sparling et al. 2018), with the latter two species not occurring in New Zealand waters. Thus, there are considerable gaps in the available information on how most New Zealand species may respond to MRED development.

Numerous international studies have identified noise pollution from pile driving during the construction of offshore wind farms as the predominant impact on marine mammals by MRED (David 2006; Madsen et al. 2006; Southall et al. 2009; Edrén et al. 2010; Teilmann and Carstensen, 2012; Thompson et al. 2013; Mortensen et al. 2021). Noise pollution from pile driving has been shown to have population-level consequences for these taxa (Teilmann and Carstensen 2012; Thompson et al. 2013; Mortensen et al. 2021). The impacts from this type of noise includes potential permanent or temporary hearing threshold shifts caused by physical injury from high-energy impulsive sounds (Madsen et al. 2006; Bailey et al. 2010; Southall et al. 2009), masking of communication or environmental cues (David 2006; Madsen et al. 2006), and behavioural disruption (Cartensen et al. 2006; Brandt et al. 2011; Vallejo et al. 2017). It has been proposed that low-frequency acoustic characteristics of baleen whales may cause these taxa to be particularly sensitive to the acoustic disturbance from marine renewable installations (Nowacek et al. 2007; Thompson et al. 2010; Bailey et al. 2014). The severity of impacts depends on: (1) the acoustic characteristics of noise, (2) proximity to source, and (3) the species present and their auditory range (Richardson et al. 1995; Madsen et al. 2006).

In Aotearoa New Zealand, the effects of pile driving noise have been demonstrated on a single species – Hector’s dolphin – during construction activity at Lyttelton Port (Leunnisen et al. 2019). The study showed the dolphins’ use of the inner harbour decreased in response to pile driving activity. Differences in the sound source and environmental characteristics mean this example may not be directly transferrable to pile driving for offshore wind installation. However, the study clearly shows that pile driving does affect the behaviour of a threatened marine mammal species.

Other than pile driving, several other impacts of MRED on marine mammals have been identified. Noise pollution from operational MREDs may have impacts on the behaviour and distribution of marine mammals (Tougaard et al. 2020; Thomsen et al. 2021). The characteristics of operational noise from offshore wind installation is highly distinct from the noise generated during the construction phase, with low-frequency specialists (e.g. baleen whales) facing higher risk (Thomsen et al. 2021). However, further work is required to explicitly demonstrate a response to operational noise. Increased vessel activity during construction, operation, and decommissioning stages is also put forward as an additional stressor that may impact marine mammals, largely due to impacts on marine mammal behaviour from both vessel presence (Constantine et al. 2015) and increased anthropogenic noise. In the US acoustic thresholds have been set that identify the level of in-air (above water) and underwater sound at which exposed marine mammals may be affected to better predict how a marine mammal’s hearing will respond to these stressors (National Marine Fisheries Service 2018). While it is yet to be determined if these thresholds and recommendations will be transferable to Aotearoa New Zealand, there will need to be considerable refinement for endemic and native species (e.g. Hector’s/Maui dolphin, NZ sea lion, NZ fur seal, southern right whale) that likely have diverse responses to noise-related stressors.

Ecological impacts on marine mammals associated with the presence (i.e. operation) of MRED have been discussed extensively internationally. Typically, ecological impacts are associated with alteration of physical (both oceanographic/seafloor) habitats or ecological processes that support foraging through prey aggregation (Bailey et al. 2014; Werner et al. 2024). Some species may also have requirements for certain habitat types for other key behaviours – e.g. resting, nursing, or breeding habitat (Weir et al. 2008; Torres et al. 2013), and thus alteration of these habitats may result in species-specific impacts. For example, southern right whales in New Zealand require shallow, sheltered, near-shore habitat for breeding and nursing young during winter (Torres et al. 2013). There is limited evidence to support long-term ecological impacts on marine mammals due to MRED presence, although research has shown displacement for multiple years post-construction is possible (Teilmann and Carstensen 2012). In contrast, other studies have demonstrated ecological benefits for marine mammals due to the presence of MRED (Scheidat et al. 2011; Russell et al. 2014), likely due to higher density of fish prey species attracted to the artificial-reef type habitat of MRED (Hammar et al. 2016; Raoux et al. 2017; Mavraki et al. 2021). For example, Russell et al. (2014) showed that harbour seals methodically use turbine pile locations to forage on aggregated prey at these locations. Additional benefits for marine mammals may include shelter/reduced disturbance from other stressors including fisheries bycatch and shipping (Scheidat et al. 2011; Hammar et al. 2016). As per pile driving impacts, the occurrence, magnitude and direction of ecological impacts on marine mammals will be species – and location-specific. With their high trophic level, marine mammals are sensitive to impacts to habitats, ecological processes and lower trophic levels that support populations of these top predators (e.g. plankton, primary and secondary production). When planning surveys to address data gaps, careful integration of data collection across each discipline/taxonomic group is important to ensure information is available to assess the broad and complex range of potential ecological responses to MRED development.

Despite the social importance of marine mammals in New Zealand, systematic surveys on the abundance, distribution and status of populations have been undertaken in only a small number of well-known locations. These include: Northland (Tezanos-Pinto et al. 2013), the Hauraki Gulf (Dwyer et al. 2016), in Maui dolphin habitat on the west coast of the Te Ika-a-Māui North Island (Constantine et al. 2021), the Te Tauihu Marlborough Sounds (Merriman et al. 2009), Kaikōura (Guerra et al. 2022), Horomaka Banks Peninsula (Brough et a., 2019), Ōtākou Otago (Turek et al 2013), Te Rua-o-te-moko Fiordland (Bennington et al. 2021) and in the sub-Antarctic (Rayment et al. 2015). Most other locations in New Zealand’s large Exclusive Economic Zone (EEZ) have a paucity of data on marine mammals that is insufficient for undertaking an appropriate assessment of the impacts of MRED on these taxa.

With the diversity of marine mammal fauna in Aotearoa and limited data, we have limited understanding of how most species will respond to marine renewable installations. This is a particular concern for our large number of threatened species (Maui and Hector’s dolphins, bottlenose dolphin, killer whale, Bryde’s whale and New Zealand sea lion). Most populations of these threatened species have limited capacity to absorb additional anthropogenic impacts (Baker et al. 2019) and so determining their likely responses to MRED should be a high priority. There is also an absence of information on the likely response of our more common coastal species (e.g. New Zealand fur seal, dusky and common dolphins, pilot whales), and migratory/seasonally present baleen whales. Internationally, studies on the latter group are particularly sparce, likely due to the low density and/or seasonal occurrence of these taxa in the areas under assessment (Thompson et al. 2010).

### Marine turtles

Marine reptiles such as turtles occur at low densities in New Zealand waters, most often being observed in northern, sub-tropical areas during the warmer months of year (Gill. 1997; Dunn et al. 2023). Of the five sea turtle species considered to occur in New Zealand waters, two species (green turtle/*Chelonia mydas* and leatherback turtle/*Dermochelys coriacea)* – are listed as ‘migrant’ under the NZTCS. However, recent research on green turtles has shown the presence of the species in New Zealand waters year-round, and indicated coastal, northern waters may be important for sub-adult individuals (Godoy et al. 2016). A foraging hotspot for leatherback turtles has also emerged in the north-east of the North Island, particularly in the Bay of Plenty (Dunn et al. 2023). The remaining three species (loggerhead turtle/*Caretta caretta,* hawksbill turtle/*Eretmochelys imbricata,* olive ridley turtle/*Lepidochelys olivacea)* are listed as ‘vagrant’ (Hitchmough et al., 2013). Under the IUCN threat classification, populations of each of the sea turtle species occurring in New Zealand waters are considered endangered (green turtle), critically endangered (leatherback, loggerhead and hawksbill turtle), and vulnerable (olive ridley turtle), due to the impact of threats on declining populations. Threats including plastic pollution, vessel strike and fisheries bycatch have been documented for green turtles in New Zealand (Godoy and Stockin 2018), and high bycatch rates of leatherbacks in pelagic fisheries in the Bay of Plenty is recognised as a key threat for that species (Dunn et al. 2023; Siders et al. 2024).

There is a paucity of information on potential impacts of MRED upon sea turtles due to a lack of overlap between these species and locations that have received the most extensive research on the impacts of MRED (i.e. the United Kingdom and the European Union) (Bailey et al. 2014). However, due to the similarity in habitat use with marine mammals, the impacts of MRED on sea turtles is often considered to be comparable (Bailey et al. 2014; Kraus et al. 2019). For example. the regular use of surface waters makes turtles highly vulnerable to vessel strike (Hazel et al. 2006; Bailey et al. 2014), which has been documented in New Zealand (Godoy and Stockin 2018). Thus, increasing vessel traffic may constitute some threat to sea turtles around MRED. Additionally, the hearing sensitivity of leatherback turtles overlaps with the acoustic characteristics of pile driving and other anthropogenic noise sources (Dow Piniak et al. 2015). Further, loggerhead turtles have demonstrated changes in diving behaviour due to noise generated from seismic surveys (DeRuiter et al. 2010). Thus, it is reasonable to conclude noise pollution from pile driving during construction of MRED may have similar impacts on sea turtles as has been documented for marine mammals.

There is currently highly limited information on the occurrence and distribution of sea turtles outside of the north-east of the North Island and thus the overlap between these species and potential MRED sites is unknown. While their occurrence in candidate MRED sites is likely low, given the high threat status for sea turtles internationally, and the impact of other threats in New Zealand (Godoy and Stockin 2018, Dunn et al. 2023), filling gaps in information on the distribution and occurrence of turtles and potential impacts of MREDs should be considered a priority. Integrating surveys of turtles with other marine megafauna (e.g. marine mammals/seabirds), would provide cost effective opportunities for filling gaps on these species.

### Seabird impacts

Collision with offshore renewable infrastructure, notably from interactions with turbine blades leading to animal injury or death, is perhaps the most high-profile of potential effects that could impact seabirds. Internationally, there is a growing and relatively substantial body of information with which to construct collision risk models that quantify risk on the basis of species, breeding population, age class, gender and season, and which link estimated mortality through collision to demographic models to better understand the magnitude of population level effects (for example, Band 2012, Lane et al. 2020, Pollock et al. 2021, Mikami et al. 2022).

Beyond direct collision, the offshore wind farm poses a potential behavioural barrier to seabirds so that part, or all, of the array area would be avoided by birds, either for foraging, when transiting to and from foraging areas or when migrating. Travelling further to forage, and therefore expending more energy, by having to navigate around a wind farm area is a potentially significant effect for adult seabirds commuting regularly from a breeding site to foraging locations to not only maintain body condition but additionally provision chicks. For example, Masden et al. (2010) calculated the additional energy expenditure for a range of seabirds associated with increasing additional distance, when foraging some distance from their breeding site. For species with relatively high wing loading (i.e. bird body mass divided by wing area) travelling an additional 10 km resulted in a 35% increase in energy expenditure (Dierschke et al. 2016). These authors also found that the two species of seabird most strongly attracted to windfarms were European shag and great cormorant, and that these species used farm infrastructure as ‘outposts’, resting on farm structures allowing birds to exploit new foraging areas relatively far offshore.

In a review of avoidance of and attraction to offshore windfarms by European seabirds, Dierschke et al. (2016) reported a range of responses, from strong avoidance through to strong attraction. It is likely that a similarly wide range of responses will be observed for seabirds in Aotearoa New Zealand. Aotearoa New Zealand supports the most diverse seabird assemblage on Earth (Croxall et al. 2012; Forest & Bird 2014a). Of the approximately 350 seabird species worldwide, 152 have been recorded from within Aotearoa New Zealand’s Exclusive Economic Zone. A total of 87 species have been recorded breeding, with nearly half (40 of 87 species, 48%) being endemic (i.e. species that breed nowhere else). Generally, seabirds in Aotearoa New Zealand are of high conservation concern: nearly 90% of all breeding species have been classified as either ‘Threatened’ or ‘At Risk’ by the NZTCS (Robertson et al. 2021). All seabird species, with a very few exceptions, are protected by the Wildlife Act (1953).

The species composition of Aotearoa New Zealand’s seabird assemblage is notably different to that in the northern hemisphere (Stephenson et al. 2021). In the northern hemisphere, the seabird fauna comprises predominantly gulls, terns and auks, with relatively few species of procellariiformes (the albatrosses, petrels and shearwaters) or shags/cormorants, although it should be noted that the north Pacific region, including the islands of the Hawaiian archipelago, supports several procellariiformes, including relatively large numbers of Australasian species during the austral winter (e.g. Shaffer et al. 2006; Rayner, Hauber, et al. 2011; Rayner, Taylor, et al. 2011; Carey et al. 2014). In Aotearoa New Zealand procellariiforme species dominate, along with penguins and shags. This difference in species composition has important implications when considering potential interactions and impacts of MRED. Specifically, while gulls, terns and auks tend to be diurnally active and generally spend the hours of darkness roosting ashore (e.g. Militão et al. 2023), many procellariiforme species are active at night (e.g. Ravache et al. 2020), which makes observing seabird activity difficult and could increase the chance of negative interactions with turbines due to low visibility conditions at night.

While there is generally good information on where seabirds breed in Aotearoa New Zealand (e.g. Forest & Bird 2014b), information on breeding population sizes and population trajectories is less comprehensive. Similarly, our understanding of how seabirds utilise marine resources, and how this varies in both space and time is reasonably good for some seabird species (e.g. Fischer et al. 2021; Thompson et al. 2021), but for the majority of species, detailed information on how seabirds utilise marine habitats is lacking. Seabirds are highly mobile and occupy relatively large, and in the case of many procellariiforme species extremely large, ranges and have widespread distributions. For example, Weimerskirch et al. (1994) showed that wandering albatrosses *Diomedea exulans* travelled as far as 3600 km from the breeding colony when foraging, a distance surpassed by the smaller white-headed petrel *Pterodroma lessonii*, which ventured as far as 5230 km from the nest site to forage during incubation (Taylor et al. 2020). Many procellariiform seabirds have overall distributions at ocean basin scales or larger (e.g. Shaffer et al. 2006; Rayner, Hauber, et al. 2011; Thompson et al. 2021). These attributes make defining the use of a specific area by seabirds, and how this use varies, extremely challenging. Traditionally, these challenges can be met by structured, systematic and temporally resolved at-sea observational surveys of seabirds in an area of interest. However, considerable resources are required to undertake such surveys and to date in New Zealand these have been completed only within relatively small spatial extents (for example, Fisher and Boren 2012; Gaskin 2017).

Finally, and importantly for any future developments of offshore windfarms in Aotearoa New Zealand, accurate and quantitative information on the flight heights of seabirds is required. These data are completely lacking which is critical to form part of an assessment of collision risk between seabirds and turbine rotors and determine potential mitigation measures (e.g. radar detection auto-activated turbine shut-down; Zehtindjiev, and Whitfield 2022). Hence, while assessments of the impacts of offshore windfarms on seabirds, or of the vulnerability of seabirds to offshore windfarms, have been completed for some jurisdictions (Garthe and Hüppop 2004; Furness et al. 2013; Reid et al. 2022), such a comprehensive assessment remains to be carried out for seabirds in Aotearoa New Zealand. To make this possible considerable effort will be required to fill present data gaps.

Despite the relative paucity of data with which to consider the potential effects of offshore renewable infrastructure on seabirds in Aotearoa New Zealand, it is nevertheless possible to infer, in a very general sense, how seabirds might be impacted. An in-depth consideration of all such effects, and how these might affect seabirds, is beyond the scope of this work and available data, but a selection of potentially impactful effects is considered here. However, it is worth reiterating that transposing information from elsewhere and applying that information within a local context is unlikely to produce realistic estimates of collision risk. This would be particularly the case for procellariforme taxa, all of which are active to some extent during the hours of darkness and the majority of which will likely vary their flight characteristics in response to weather conditions, notably wind speed.

Should MRED proceed in Aotearoa New Zealand, monitoring of seabird collisions once turbines become operational poses several challenges. Physical recovery of collision victims is impractical, but elsewhere camera technology has been used to assess the occurrence of collisions within the wind turbine array (Tjørnløv et al. 2023), and within-blade sensor technology could prove useful in characterising collisions (Clocker et al. 2021).

Offshore renewable technology infrastructure could result in several positive outcomes for seabirds. As noted above, artificial reef effects of hard infrastructure could increase species richness and abundance of potential seabird prey. Where the MRED development acts as a marine reserve, with minimal or zero extraction of marine resources from within the turbine area, enhanced levels of seabird prey abundance might extend throughout the wind turbine array more generally, and not just in association with individual turbines. This could potentially represent a predictable (i.e. fixed in space) and enhanced food supply for those seabird species able to take advantage of such resources. The near-field physical disturbances can also affect seabird foraging behaviour as it changes circulation patterns in the water wake of a turbine (Lieber et al. 2019). Additionally, renewable technology infrastructure could be utilised by seabirds as resting and roosting platforms. For species such as shags, which are relatively poor flyers but accomplished divers (Watanabe et al. 2011), the provision of physical structures would allow shags to rest and dry feathers (shags do not have water-repellent plumage typical of other seabirds) when at sea between foraging bouts, effectively extending foraging trips and the likelihood of capturing more prey items before having to return to nest sites or roost sites ashore. Dierschke et al. (2016) reported two species of cormorant as being highly attracted to European windfarms, using farm infrastructure as ‘outposts’, allowing birds to exploit new foraging areas relatively far offshore.

## Future perspectives

### MRED positives and opportunities

There are likely many positive outcomes from MRED development discussed in the previous sections, over and above carbon emissions mitigation, most of which revolve around exclusion of other activities (Carter et al. 2014) and the insertion of new hard substrate structures into the marine environment. In particular, positive effects have been recorded for commercial and recreational fisheries (Watson et al. 2024; Werner et al. 2024).

As initial offshore wind developments take place there is a significant opportunity to enhance these positive aspects with MRED structures and configurations designed to meet conservation needs (Werner et al. 2024). As New Zealand uptake of MRED is relatively late compared to other countries, we can leverage research and tested methodologies used elsewhere for all stages of the MRED lifecycle. For example, opportunities will evolve from wind-based MRED for development of tidal and wave energy extraction initiatives (e.g. Majdi Nasab et al. 2021). The technology exists for these alternate sources and could become economically viable if wind enables sufficient development of marine operations (Wilberforce et al. 2019). These approaches have overlapping and unique impacts relative to offshore wind (Copping et al. 2020). This would have the benefit of accessing a more diverse array of energies in terms of resource timing as well as providing opportunities for co-location (Weiss et al. 2018).

In addition, during the MRED scoping and impact assessment process, the collection of marine data in data-poor regions will also have wider benefits. Much of Aotearoa’s offshore shallow continental shelf water column and benthic systems are under-studied and under-sampled (Gordon et al. 2010; Rowden et al. 2012; Stevens et al. 2021). Here we have maintained a primary focus on the Taranaki region identified by MBIE (2022) as there is more known about the region than the other targeted areas. This is despite the proximity of the west Waikato coastal zone to the major urban centre of Tāmaki Makaurau Auckland, and despite the significant energy-using aluminium refinery in Bluff just to the north of the Te Ara a Kiwa Foveaux Strait area (Figure 1). Of course, due to commercial sensitivities these new data will potentially not be openly disseminated.

As an example of data paucity, soft-sediment habitats, which typically have diverse associated biotic assemblages (Rowden et al. 2012), are suitable for offshore renewable infrastructure development but are typically poorly documented in Aotearoa New Zealand. While offshore infrastructure surveys in the South Taranaki Bight have added to current knowledge of soft sediment habitats (e.g. Anderson et al. 2015; Beaumont et al. 2015), information on shallower sedimentary environments within the Horizons coastal marine area is still sparse (Hale et al. 2021). As such, pre-infrastructure establishment environmental impact assessment surveys and subsequent monitoring for effects can provide key additional information in the currently understudied regions of the New Zealand EEZ proposed for MRED. Additionally, with the use of renewable technology infrastructure by seabirds as resting and roosting platforms, valuable observations of species movements and abundance could also be taken.

### Vulnerabilities and knowledge gaps

There is clearly a wide-ranging lack of data for almost all relevant metrics in Aotearoa New Zealand compared to, for example, Europe or the USA. As well as a small economy, the energetic marine environment makes for difficult surveying conditions. Traditional vessel-based, aerial and acoustic survey platforms are challenged by ocean and weather conditions and thus future data collection may include emerging new technologies (i.e. autonomous marine and aerial vehicles) to collect baseline data and regularly monitor and manage impacts of MRED both temporally and spatially on impacted habitats and vulnerable species.

Lifetime perspectives of MRED activities should take into account marine sediment habitat ecosystem services and the impacts of MRED development and operation on these are not well characterised (De Borger et al. 2021; Watson et al. 2024) A recent stocktake of New Zealand organic carbon stocks in marine sediments shows an uneven distribution of these stocks across Aotearoa New Zealand, with the current planned regions for development having a relatively low organic carbon content (only a few percent) compared to other regions (Nodder et al. 2023). The disturbance of such sediment repositories, however, has the potential to release remineralised carbon back into the water column, with possible implications for carbon emissions (Atwood et al. 2024). Overall effects may be location and context specific. Despite the initial disturbance, potential increased productivity as a result of the MRED presence could be a net positive for benthic organic carbon accumulation (De Borger et al. 2021), or in-water structures may break down stratification, reduce nutrient upwelling, and decrease pelagic productivity in some areas, particularly downstream (e.g. Dorrell et al. 2022).

The various knowledge gaps compound uncertainty for holistic approaches such as Ecosystem Based Management which provides an understanding of the non-linear interactions between all aspects of the system. For example, reduction in fishing activity observed around offshore windfarms in locations overseas provides opportunities for co-location of other commercial developments (Watson et al. 2024) or marine protected areas (Dunkley and Solandt 2022). Alternatively, increased spacing between turbines or structures (> 1 nm) which allows fishing in-between can allow fisheries to take advantage of increased fish biomass within MRED zones (Drew et al. 2024).

The cumulative effects of MRED on the marine environment should be considered alongside concurrent effects of climate change during infrastructure planning stages and take into account all life stages of the project. Specific and comprehensive assessment of the risks to marine ecosystems and processes at proposed offshore infrastructure installation sites will be vital to ensure that the offshore energy sector is environmentally sustainable (Galparsoro et al. 2022) now and in the future. Following the establishment of an array of MRED, the progressive development of further offshore energy production types with expansion into deeper areas and further offshore will require subsequent assessment as greater development in this offshore region increases space-user interactions (Galparsoro et al. 2022), cumulative effects (Gușatu et al. 2021) and impacts on additional processes (Dorrell et al. 2022).

Standardised monitoring protocols for all renewable near – and off-shore infrastructure incorporating suitable metrics with respect to the benthos and benthic communities will enable identification of changes to habitat quality, community composition and ecosystem function or service provision (Wilding et al. 2017). Monitoring recommendations for known and new non-indigenous species incursions will require cost-effective surveillance which may require validation and operation of passive monitoring technologies including autonomous video capture or environmental DNA monitoring technologies (Tait et al. 2018; von Ammon et al. 2018; Dahlgren et al. 2023).

To ensure that offshore renewable developments do not adversely impact cultural values, it is crucial to identify taonga (treasured) species of interest to local iwi and hapū, as well as the habitats of particular significance for these species. It is also important to identify and avoid traditional fishing grounds, wāhi tapū (sacred sites) and access points to these places to ensure local iwi and hapū can still take part in their cultural practices. In the Aotearoa context, there is an opportunity for MRED to create environmental monitoring programmes that use Māori monitoring methods and indicators alongside western scientific ones (e.g. Moller et al. 2004; Crow et al. 2020). This will enable consideration of environmental impacts through a holistic whole-of-socio-environment lens.

### Te Tiriti implications

There is a growing body of literature that discusses Māori and indigenous rights in relation to ownership and equitable transitions to renewable energy (e.g. Berka et al. 2020). The New Zealand Climate Change Commission states that for an equitable transition to a low emissions future for Māori, Māori should be enabled to participate in decision-making and provided with support to build capability in the renewable energy space (He Pou a Rangi, 2021). However, little investigation has been done on Māori perspectives of the environmental implications of MRED. It is important to recognise the need to refer to the tikanga (laws, customs) of the local people, as ideas and practices differ from one tribal region to the other (Mead 2003). Iwi and hapū environmental plans and cultural impact assessments are two types of sources that can provide some clarity about local Māori perspectives in absence of formal relationships.

Iwi and hapū environmental management plans (IEMPs) are developed by hapū or iwi to identify environmental kaupapa (agendas, issues) of significance and details around how they expect to engage in environmental planning and decision-making processes. These IEMPs can vary in style, content, spatial and temporal specificity – and can include outcomes sought, concerns, issues, objectives, methods and/or policies in relation to various environmental kaupapa. The plans are lodged with local and regional councils to be primarily used as planning documents considered during consent processes and where activities may impact the relevant iwi/hapū rohe (area, territory).

For example, in regard to coastal environmental impacts of offshore development, a cultural impact assessment of an aquaculture proposal in the Foveaux Strait identified a number of adverse effects beyond the usual issues to avoid (Tipa 2020). These included kai (food) species, loss of habitats for taonga species, loss of wāhi tapū (restricted sites) and wāhi taonga (treasured sites). Development and examination of Iwi and hapū environmental management and cultural impact assessment plans does not replace direct engagement with the relevant iwi/hapū to obtain their input – but it does include useful background information to inform future strategies and discussions with iwi and hapū.

### Closing thoughts

It is clear that mitigation of greenhouse gas emissions needs to be a central pillar of every national response to the global climate emergency and so expanding the use of renewable energy resources is a natural and sensible consequence. Environmental impacts in the context of the changing climate face a challenge in that the baseline against which (acceptable) impact is measured is, in some instances, shifting with the climate. The challenge is exacerbated in that the shifting baseline is not due to the singular activity in question, but to all human contributions to carbon emissions. It is certain that having accessible and interpretable data and associated understanding will be central to making informed decisions in the marine space. Development of MRED in Aotearoa New Zealand poses some unique challenges around incorporation of te ao Māori and Te Tiriti principles, protection of key taonga, endemic, and vulnerable species, particularly seabirds and marine mammals, that migrate through and utilise the coastal regions, and understanding the underlying biogeophysical changes that could occur with this activity. Understanding and mitigating negative effects will be essential for an equitable and sustainable development of Aotearoa New Zealand’s marine renewable energy sector, while the positive effects of MREDs present new opportunities for enhancement of the potential benefits to society.
